# Left Atrial Fibroelastoma as a Cause of Stroke: A Case Report

**DOI:** 10.3390/medicina58020182

**Published:** 2022-01-26

**Authors:** Jing-Chun Lin, Szu-Yu Tsai, Ta-Shen Kuan, Wei-Pin Lin

**Affiliations:** 1 Department of Physical Medicine and Rehabilitation, National Cheng Kung University Hospital, College of Medicine, National Cheng Kung University, Tainan 704, Taiwan; tiff831020@gmail.com (J.-C.L.); Lac0417@gmail.com (S.-Y.T.); kuan@mail.ncku.edu.tw (T.-S.K.); 2 Department of Physical Medicine and Rehabilitation, College of Medicine, National Cheng Kung University, Tainan 704, Taiwan

**Keywords:** cardiac papillary fibroelastoma, stroke, left atrium

## Abstract

Cardiac papillary fibroelastoma is a benign and rare primary tumor of the heart that is most frequently located in the aortic or the mitral valves. Papillary fibroelastoma arising from the left atrium is exceedingly rare, comprising less than 7% of all cardiac papillary fibroelastomas. Tumors in this location could be a source of cardioembolic stroke, often in the anterior circulation of the cerebrum. A 66-year-old female presenting with right hemiparesis, central facial palsy, homonymous hemianopia, and dysarthria received intravenous thrombolysis for stroke without apparent improvement. Magnetic resonance imaging of the brain revealed ischemic infarction in the territories of the left middle and posterior cerebral arteries. A tumor with a maximal diameter of 2.3 cm was disclosed during workup for possible cardioembolic stroke with transthoracic echocardiography and computed tomography of the heart. The clinical course was complicated by stroke-in-evolution and hemorrhagic transformation. The patient underwent left atrial tumor excision and left atrium appendage closure. In-patient stroke rehabilitation programs were also initiated after the conditions stabilized. No clinically significant complications developed after the operation. Neurological functions improved and the patient was able to perform most basic daily living activities with some assistance. An exhaustive search for the cause of cardioembolic stroke is paramount, as management strategies may differ from patients with thrombotic stroke.

## 1. Introduction

The etiology of ischemic stroke can be broadly categorized into five subtypes: large-artery atherosclerosis, cardioembolism, small-vessel occlusion, stroke of other determined etiology, and stroke of undetermined etiology [1]. In cases caused by cardioembolism, a thorough investigation for the cause and nature of the emboli is vital as it often alters the treatment strategy. Cardiac papillary fibroelastoma is a benign and rare primary tumor of the heart that can occasionally cause cardioembolic stroke. Most of the tumors arise from the aortic and the mitral valves, with left atrial tumors being exceedingly rare. Prompt surgical intervention is warranted to prevent further embolic events [2].

## 2. Case Presentation

**Figure 1:**
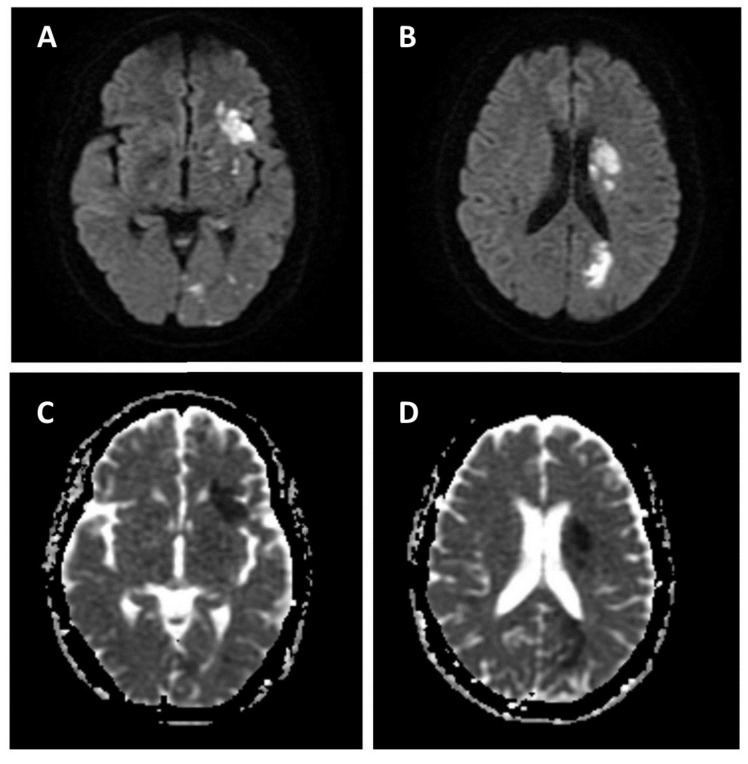
Brain MRI showing scattered ischemic infarction in the left middle cerebral and posterior cerebral artery territories. (**A**,**B**) Diffusion-weighted imaging; (**C**,**D**) apparent diffusion coefficient.

**Figure 2:**
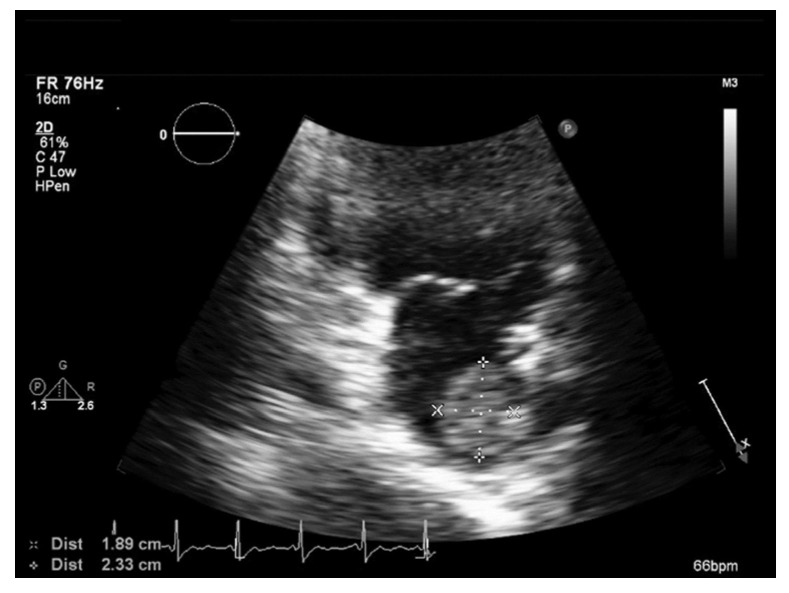
Transthoracic echocardiography revealing a 1.89 × 2.33 cm mass lesion in the left atrium.

**Figure 3:**
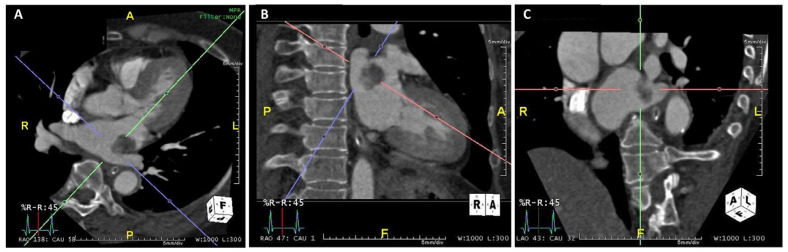
CT of the heart with contrast medium confirmed the presence of a left atrial pedunculated lobulated soft tissue nodule abutting the left lateral wall with internal migration into the left inferior pulmonary vein, measuring 2.3 cm in its greatest dimension. (**A**) Axial view; (**B**) sagittal view; (**C**) coronal view.

A 66-year-old female (weighing 50.3 kg) with hypertension and hypothyroidism under amlodipine and levothyroxine presented with acute right hemiparesis, right mouth angle drooping, and absent verbal output. She arrived at the hospital approximately 2 h after the onset of symptoms, with the initial National Institutes of Health Stroke Scale (NIHSS) being 14. Neurological examination disclosed aphasia, right central facial palsy, right hemiparesis, and positive Babinski sign on the right side. No evidence of intracranial hemorrhage was discovered on computed tomography (CT) of the brain, and the initial laboratory results were all within normal limits. The patient received intravenous recombinant tissue plasminogen activator with a dose of 0.9 mg/kg, 45 mg in total. CT perfusion scan and angiography revealed a wedge-shaped hypoperfusion area in the left frontal region with occlusion of the left superior distal M2 segment of the middle cerebral artery, at its bifucation into the M3 segment. Endovascular thrombectomy was not performed as the occlusion site was too distal for the procedure. Motor status of the right limbs deteriorated from hemiparesis to hemiplegia during the first two days of hospitalization, without demonstrable hemorrhagic transformation on repeated CT scan. Magnetic resonance imaging (MRI) of the brain disclosed scattered ischemic infarction in the left middle cerebral and posterior cerebral artery territories (Figure 1). A mass lesion in the left atrium was discovered via transthoracic echocardiography (TTE) during etiologic survey for possible cardioembolic stroke, with the morphology indicative of a tumor (Figure 2). CT of the heart with contrast medium confirmed the presence of a left atrial pedunculated lobulated soft tissue nodule abutting the left lateral wall with internal migration into the left inferior pulmonary vein, measuring 2.3 cm in its greatest dimension (Figure 3).

**Figure 4:**
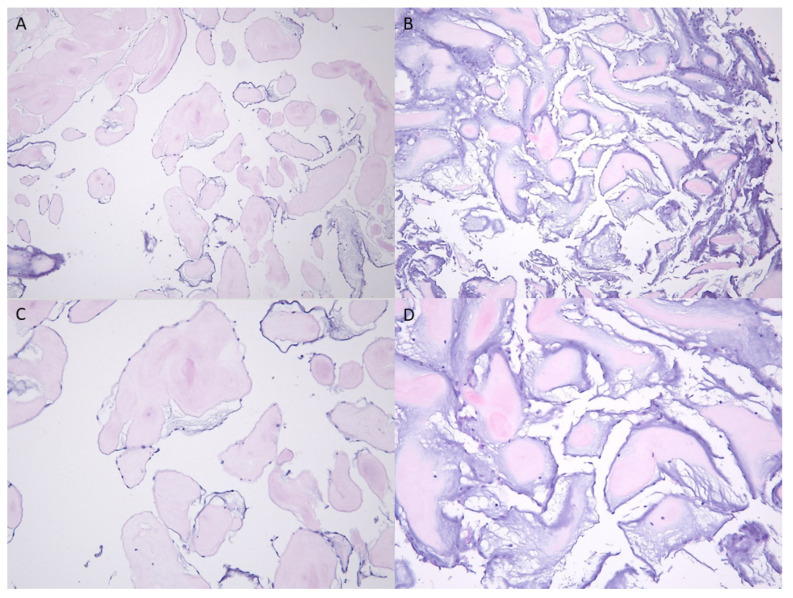
Histopathology of the specimen with H&E stain revealed branching papillary fronds of central avascular collagen with marked myxoid stroma and variable elastic tissue lined by hyperplastic endothelial cells. (**A**,**B**) 100×; (**C**,**D**) 200×.

On the fourth day, motor function of the right limb continued to worsen, and repeated CT of the head revealed a new wedge-shaped infarct in the left temporal region with hemorrhagic transformation. The patient underwent left atrial tumor excision and left atrial appendage closure smoothly on the sixth day. Grossly, the mass resected from the left atrium was a 2.5 × 2.0 × 0.4 cm fragile tumor adhering to the orifice of the left inferior pulmonary vein. Histopathologic examination of the specimen revealed branching papillary fronds of central avascular collagen with marked myxoid stroma and variable elastic tissue lined by hyperplastic endothelial cells (Figure 4). The histopathologic findings were consistent with papillary fibroelastoma. Post-stroke rehabilitation programs were initiated after stabilization of her conditions, with standard precautions for patients with sternotomy and heart surgery. Aspirin was added for secondary stroke prevention after repeated CT of the head revealed stabilization of stroke lesions. Motor function of the right lower extremity improved to stage IV on the Brunnstrom staging, but gross synergistic pattern persisted in the right upper limb. Language function also improved during the course of in-patient rehabilitation, with good comprehension, reading and writing ability, and the persistence of mild apraxia of speech. Upon discharge from hospital, the patient still required minimal assistance for ambulation and minimal to moderate assistance for most daily living activities.

## 3. Discussion

Multiple risk factors have been identified for cardioembolic stroke, the most common of which include atrial fibrillation, systolic heart failure, recent myocardial infarction, patent foramen ovale, prosthetic heart valves, and infective endocarditis [3]. Other rarer causes include papillary fibroelastoma, myxoma, and mitral calcification, each accounting for less than 1% of all cardioembolic strokes [3]. As a consequence of such a wide variety of conditions that can cause cardioembolic stroke, probing into the possible contributing factor is crucial for optimizing patient care. For all patients with ischemic stroke, cardiological evaluation, including cardiovascular history, electrocardiography, chest X-ray, and echocardiography, should be considered. In patients with presumed cardioembolic stroke, transthoracic echocardiography (TTE) is usually the first-line imaging modality for evaluation of the possible source of emboli. Transesophageal echocardiography, on the other hand, can be ordered for patients with negative TTE and high clinical suspicion of intracardiac thrombi. Those imaging studies are critical as the finding of intracardiac thrombi could dictate future treatment plans [4].

Cardiac papillary fibroelastomas (CPFs) were previously believed to be less common than cardiac myxomas. The mean age of diagnosis is at 60 years, without a gender preference. Valvular CPFs, on the other hand, have been reported to develop in patients younger than 30 years of age [2]. With increasing echocardiography surveillance, recent studies indicate that CPFs may actually be more common than cardiac myxomas [5]. CPFs have a propensity to develop on the aortic and mitral valves and rarely tether to the atrial epithelium, with tumors located in the left atrium accounting for just 6.1% of all tumors [2]. Reviewing the English language literature reporting cases of left atrial fibroelastomas (Table 1), the most common origin of those tumors is the left atrial appendage (39.3%) and the left atrial ridge (36.3%). Only a single case of fibroelastoma originating from the lateral wall has been reported [6]. Transesophageal echocardiography has been recommended in suspected cases if routine transthoracic echocardiography fails to detect a tumor, as the sensitivity and specificity of the latter procedure decrease with tumors measuring less than 20 mm^3^ [2].

CPFs are believed to be an acquired condition. Postulated contributing factors include epithelial hyperproliferation from hemodynamic trauma, organized thrombi, underlying genetic predisposition, prior cardiac procedures, radiation therapy, and chronic cytomegalovirus endocarditis [2]. None of these factors are identified in this present case. Given the fragile composition of these tumors, segments of the tumor could dislodge and lead to systemic embolization. Additionally, the mucopolysaccharide and hyaluronic-acid-rich frond surface may harbor thrombi that later embolize. In cases of cardioembolic stroke caused by thrombi originating from the surface of CPFs, treatment with intravenous (IV) thrombolysis can be a viable option. On the other hand, cardioembolic strokes from segments of the tumor proper might be resistant to thrombolytic therapy [7], likely explaining the limited effect of thrombolytic therapy on our case. Despite this, patients with CPF-related cardioembolic stroke should not be excluded from receiving thrombolytic therapy. In a retrospective study of 725 cases of CPF, thrombi were reported in 19 cases perioperatively, and 3 cases of recurrent strokes were noted despite warfarinization [8].

Large retrospective analyses estimate that the risk of stroke or transient ischemic attacks in patients with CPF is between 13.5% and 53.6% [2]. At a mean follow-up of 31 months, between 16% and 24% of patient with nonresected tumors developed recurrence of stroke [2]. CPF at the left side of the heart demonstrated a higher potential of embolic events [9]. Among the 33 cases with left atrial CPF reported in the literature (Table 1), 54.5% suffered from cerebral vascular accidents, including 39.3% with stroke and 15.1% with transient ischemic attacks. In contrast, the risk of pulmonary embolism from right atrial CPF is low. Only 2 cases presented with pulmonary embolism in a review of 17 cases with right atrial CPF [10]. Most right atrial CPFs were discovered incidentally during survey of unrelated symptoms or signs [10]. The reason for the different risks of embolic events between right and left atrial CPF is not clear so far. Low rates of stroke recurrence and excellent survival rates were reported after resection of the tumor [11]. Although the recurrence rate of CPF is low, the location of recurrence might be different from the original tumor [12]. A 10-year follow-up study of 12 patients with CPF reported no evidence of tumor recurrence via TTE. Among the seven patients who received TEE during the follow-up period, however, recurrence was detected in two patients [12]. Therefore, TEE could be superior to TTE as the follow-up tool for patients with CPF, and careful screening of all heart chambers beside the original tumor location is critical. To date, no consensus exists on the appropriate anticoagulation regimen after tumor resection, but anticoagulation should be considered if surgery is not an option [13].

## 4. Conclusions

Cardiac papillary fibroelastoma is a benign cardiac tumor that can cause cardioembolic stroke. Exhaustive search for the cause of cardioembolic stroke is paramount to direct management strategies and optimize patient care.

## Figures and Tables

**Table 1:** Cases of left atrial fibroelastoma reported in the English language literature.

Year	Authors	Age	Sex	Symptoms	Size	Origin	Year	Authors	Age	Sex	Symptoms	Size	Origin
1994	Nakao et al.	60	F	Incidental	8 mm	Septum	2009	Bicer et al.	72	M	Stroke	1.2 × 0.8 cm	MW
1997	Klarich et al.	NA	NA	NA	NA	NA	2010	Atalay et al.	70	M	NA	3 × 2.6 × 2.9 cm	LAA
1999	Howard et al.	61	M	TIA	20 mm	Ridge	2011	Bhat et al.	53	F	Pectoralis angina	1 cm × 2 cm	LAA
2000	Tsukube et al.	47	M	Incidental	15 × 70 mm	LAA	2011	Chen et al.	59	F	TIA	1.5 × 1 cm	LAA
2001	Wolber et al.	63	F	TIA	27 × 25 × 22 mm	Ridge	2013	Saitoh et al.	78	F	Incidental	17 × 14 × 10 mm	Ridge
2001	Friedman et al.	84	F	Spinal stroke	10 mm	Ridge	2014	Waziri et al.	70	F	MI	0.9 × 1.5 cm	Ridge
2001	Sidhu et al.	59	F	Stroke	15 mm	LAA	2015	Oda et al.	49	M	Stroke	8 mm × 8 mm	Ridge
2002	Gowda et al.	74	M	Stroke	10 × 15 mm	Ridge	2017	Cook et al.	64	M	TIA	1 × 0.7 cm	Ridge
2004	Butany et al.	63	F	Incidental	24 × 20 mm	Septum	2019	Bonavia et al.	76	M	Stroke	22 × 18 × 12 mm	LAA
2005	Perzanowski et al.	63	F	Incidental	NA	LAA	2019	Mashicharan et al.	72	M	Stroke	0.7 × 0.7 cm	Ridge
2007	Idahosa et al.	79	M	TIA	15 × 15 mm	Ridge	2019	Roberts et al.	71	F	Stroke	1.2 × 0.7 cm	LAA
2007	Mohammadi et al.	59	M	Stroke	5 × 5 mm	LAA	2019	Smith et al.	41	F	NA	NA	NA
2007	Shimode et al.	76	M	Incidental	10 × 7 mm	LAA	2020	Tsugu et al.	70	F	Incidental	34 × 8 × 3 mm	Septum
2008	Barcena et al.	76	F	Stroke	5 × 5 mm	LAA	2020	Vieira et al.	71	F	Progressive fatigue	1.6 × 1.3 cm *	LW
2008	Jablonski- Cohen et al.	51	F	Incidental	2 × 2 cm	LAA	2021	Alozie et al.	58	M	Stroke	8 × 9 mm	LAA
2009	Hirose et al.	67	M	Stroke	10 × 10 mm	Ridge			75	M	Stroke	13.5 × 12.7 mm **	Ridge
							2021	Current	66	F	Stroke	2.3 cm	LW

F, female; M, male; NA, not available; TIA, transient ischemic attack; LAA, left atrial appendage; MI, myocardial infarction; LW, lateral wall; MW, medial wall; * three left atrial masses were found, largest one measured 1.6 × 1.3 cm; ** two left atrial masses were excised, second mass: 5 mm.

## Data Availability

The data presented in this study are available on request from the corresponding author. The data are not publicly available owing to privacy.
